# Low birth weight and its associated factors in Ethiopia: a systematic review and meta-analysis

**DOI:** 10.1186/s13052-018-0586-6

**Published:** 2018-11-26

**Authors:** Aklilu Endalamaw, Eshetu Haileselassie Engeda, Daniale Tekelia Ekubagewargies, Getaneh Mulualem Belay, Mekuriaw Alemayehu Tefera

**Affiliations:** 1 Department of Pediatrics and Child Health Nursing, School of Nursing, College of Medicine and Health Sciences, University of Gondar, Gondar, Ethiopia; 2 Department of Environmental and Occupational Health and Safety, Institute of Public Health, College of Medicine and Health Sciences, University of Gondar, Gondar, Ethiopia

**Keywords:** Low birth weight, Birth outcome, Ethiopia

## Abstract

**Background:**

Different primary studies in Ethiopia showed the burden of low birth weight. However, variation among those studies was seen. This study was aimed to estimate the national prevalence and associated factors of low birth weight in Ethiopia.

**Methods:**

PubMed, Web of Science, Cochrane library, and Google Scholar were searched. A funnel plot and Egger’s regression test were used to see publication bias. I-squared statistic was applied to check heterogeneity of studies. A weighted inverse variance random-effects model was applied to estimate the national prevalence and the effect size of associated factors. The subgroup analysis was conducted by region, study design, and year of publication.

**Result:**

A total of 30 studies with 55,085 participants were used for prevalence estimation. The pooled prevalence of LBW was 17.3% (95% CI: 14.1–20.4). Maternal age < 20 years (AOR = 1.7; 95% CI:1.5–2.0), pregnancy interval < 24 months (AOR = 2.8; 95%CI: 1.4–4.2), BMI < 18.5 kg/m^2^ (AOR = 5.6; 95% CI: 1.7–9.4), and gestational age < 37 weeks at birth (AOR = 6.4; 95% CI: 2.5–10.3) were identified factors of LBW.

**Conclusions:**

The prevalence of low birth weight in Ethiopia remains high. This review may help policy-makers and program officers to design low birth weight preventive interventions.

## Background

Globally, low birth weight (LBW) is one of the major public health problems of newborns [1] that predispose newborns to many health disorders, like hypoglycemia [2], hypothermia [3], mental retardation, physical, and neurodevelopmental problems [4, 5]. Consequently, the risk of death is high in LBW infants. According to WHO’s 2014 estimation, 4.53% of total deaths in Ethiopia was due to LBW [6].

Globally, 15 to 20% of newborns were LBW [7]; 13% in sub-Saharan Africa [7], and 15.9% in ten developing countries (Armenia, Cambodia, Colombia, Indonesia, Jordan, Nepal, Pakistan, Tanzania, Uganda, and Zimbabwe) [8]. As a result, LBW executes a remarkable burden on the political, social, economic, and healthcare system both in developing and developed nations. Hence, by the end of 2025, World Health Assembly set a policy target to reduce LBW by 30% [7]. Strategies have been implemented to reduce newborn with below normal birth weight with given emphasis on the packages of care provided at the prenatal, ante-natal, intra-natal, and post-natal period.

Despite these preventive strategies, many studies in different settings of the world revealed the contributing factors of LBW as the maternal [9–16], prenatal [17–21], delivery and neonatal [21–25], helminthic and parasitic-related factors [26–28]. Additionally, HIV-positive pregnant women who were on advanced WHO clinical stage of AIDS and having a lower CD4 cell count [26] were more likely to deliver below normal birth weight baby.

Similarly, in Ethiopia, globally recommended strategies have been implemented. Variety of studies were conducted to estimate the prevalence of LBW in this country. However, prevalence of LBW ranges from 8% [29] to 55.9% [30] which showed a great variation across different geographical settings and different time periods. Besides, there is no nationally represented pooled data of LBW in Ethiopia. Therefore, this systematic review and meta-analysis was aimed, firstly, to estimate the pooled prevalence of low birth weight and secondly, to estimate the effect size of associated factors of LBW in Ethiopian context.

## Methods and materials

### Reporting

The results of this review were reported based on the Preferred Reporting Items for Systematic Review and Meta-Analysis statement (PRISMA) guideline [31] (Supplementary file-PRISMA checklist) and, it is registered in the Prospero database: (PROSPERO 2017: CRD42017074407) Available from http: // www. Crd. york. ac. uk/ PROSPERO_REBRANDING/ display_record. asp? ID = CRD42017074407.

### Inclusion and exclusion criteria

Cross-sectional, case-control, and cohort studies were included. Those studies had reported the prevalence and/or at least one associated factors of LBW and published in English were considered. There was no restriction of the study period. Citations without abstract and/ or full-text, anonymous reports, editorials, and qualitative studies were excluded from the analysis.

### Searching strategy and information sources

PubMed, Web of Science, Cochrane library, and Google Scholar were accessed. Articles with incomplete reported data were handled through contacting corresponding authors. The core search terms and phrases were “newborn”, “neonate”, “abnormal birth weight”, “birth outcome”, “underweight”, “low birth weight”, “Ethiopia”. The search strategies were developed using different Boolean operators. Notably, to fit advanced PubMed database, the following search strategy was applied: [(newborn [MeSH Terms] OR neonate OR infant OR child OR children AND (birth weight [MeSH Terms] OR birth outcome OR low birth weight OR very low birth weight OR underweight OR macrosoma OR big baby weight OR normal birth weight OR abnormal birth weight) AND (Ethiopia)].

### Study selection

Retrieved studies were exported to reference manager software, Endnote version 7 to remove duplicate studies. Three independent reviewers screened the title and abstract. The disagreement was handled based on established article selection criteria. Three independent authors conducted the abstract and full-text review.

### Quality assessment

Three independent authors appraised the quality of studies. The Joanna Briggs Institute (JBI) quality appraisal checklist was used [32]. The disagreement was resolved by the interference of third reviewer. The following items were used to appraise cohort studies: [1] similarity of groups, [2] similarity of exposure measurement, [3] validity and reliability of measurement, [4] identification of confounder, [5] strategies to deal with confounder, [6] appropriateness of groups/participants at the start of the study, [7] validity and reliability of outcome measured, [8] sufficiency of follow-up time, [9] completeness of follow-up or descriptions of reason to loss to follow-up, [10] strategies to address incomplete follow-up, and [11] appropriateness of statistical analysis. The items used to appraise case-control studies were: [1] comparable groups, [2] appropriateness of cases and controls, [3] criteria to identify cases and controls, [4] standard measurement of exposure, [5] similarity in measurement of exposure for cases and controls, [6] handling of confounder [7], strategies to handle confounder, [8] standard assessment of outcome, [9] appropriateness of duration for exposure, and [10] appropriateness of statistical analysis. Studies got 50% and above of the quality scale were considered low risk. The following items were used to appraise cross-sectional studies: [1] inclusion criteria, [2] description of study subject and setting, [3] valid and reliable measurement of exposure, [4] objective and standard criteria used, [5] identification of confounder, [6] strategies to handle confounder, [7] outcome measurement, and [8] appropriate statistical analysis. Studies were considered low risk when it scored 50% and above of the quality assessment indicators.

### Data extraction

Two independent reviewers extracted data using a structured data extraction form. Whenever variations of extracted data observed, the phase was repeated. If discrepancies between data extractors continued, third reviewer was involved. The name of the first author and year, the study region, the study design, the target population, the sample size, prevalence of LBW, and AOR of associated factors were collected.

### Outcome measurement

LBW was considered, when newborn weight recorded below 2500 g [33].

### Statistical analysis

Publication bias was checked by funnel plot and more objectively through Egger’s regression test [34]. Heterogeneity of studies was quantified using the I-squared statistic, in which 25, 50, and 75% represented low, moderate and high heterogeneity respectively [35, 36]. Pooled analysis was conducted using a weighted inverse variance random-effects model [37]. Subgroup analysis was done by the study region, design, and year of publication. Sensitivity analysis was employed to see the effect of single study on the overall estimation. Besides, the time-trend analysis was conducted to check whether variations through time is observed. STATA version 14 statistical software was used for meta-analysis.

## Result

### Characteristics of included studies

**Fig. 1:**
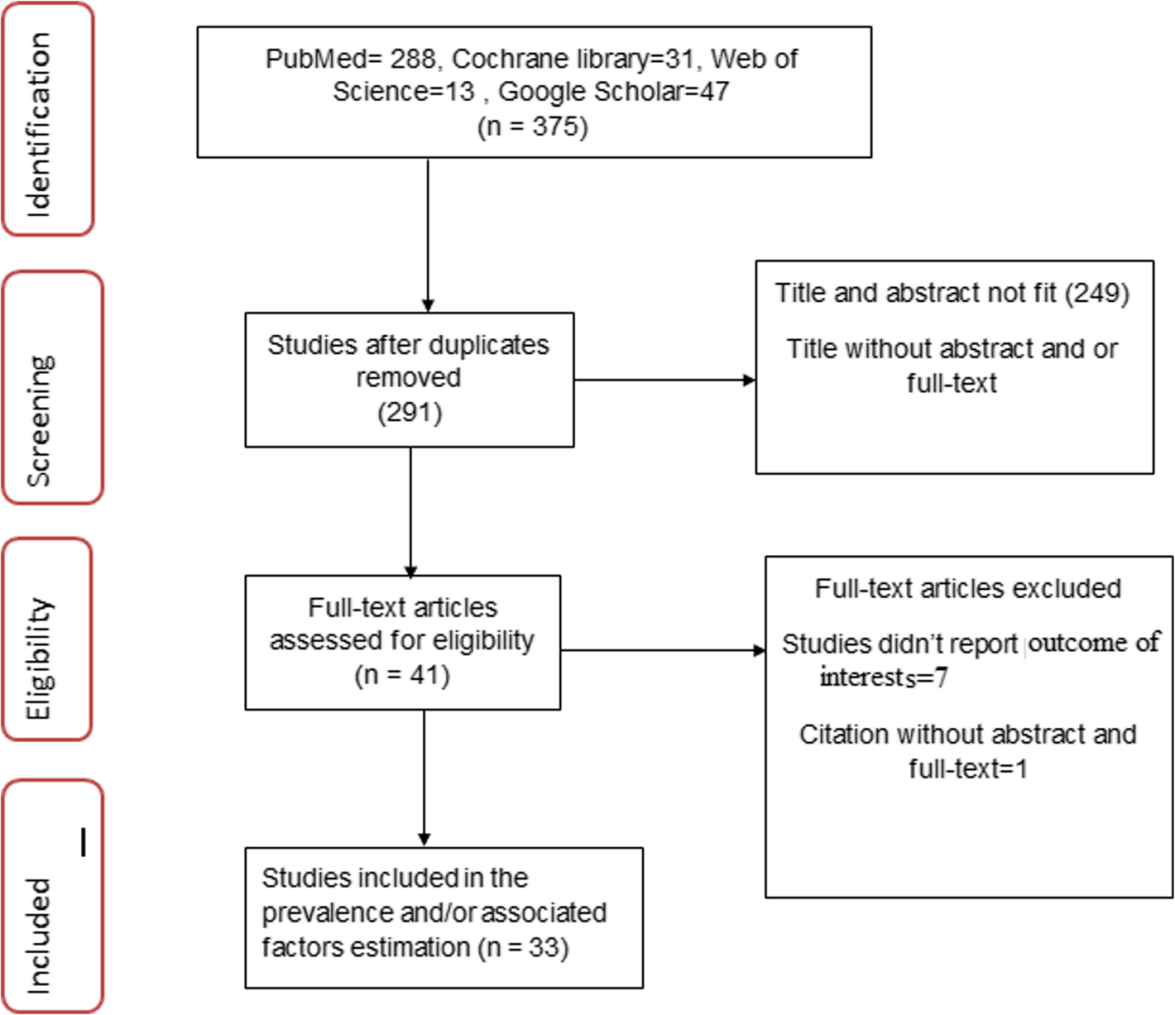
The study selection process

The search strategy retrieved 288 from PubMed, 31 from Cochrane library, 13 from Web of Science, and 47 from Google Scholar. After duplication removed, 291 remained. Finally, 41 studies were screened for full-text review and 33 were to the prevalence and/ or associated factors analysis (Fig. 1).

Nine studies were found in Amhara region [38–46], ten in Oromia [47–56], six in Tigray [57–62], five in Addis Ababa [30, 63–66], and three in Southern Nation Nationalities and Peoples region (SNNPR) [29, 67, 68]. Twenty-two of the studies were done by cross-sectional study design, three studies by case-control study design, whereas eight of the studies were conducted through cohort study design respectively. Regarding year of publication, six studies were published before 2000, seven studies were published between 2000 and 2010, and 20 studies were between 2010 and 2017 (Table 1).

**Table 1:** Characteristics and quality status of the studies

First Author/Year	Study region	Study design	Sample size	Prevalence (%)	Quality status
Zenebe et al./2014 [38]	Amhara	Cross-sectional	540	22.5	Low risk
Gebremariam A/2005 [47]	Oromia	Cross-sectional	1441	10.2	Low risk
Abdo RA et al./2016 [67]	SNNPR	Cross-sectional	327	9.8	Low risk
Adane AA et al./2014 [39]	Amhara	Cross-sectional	481	11.2	Low risk
Assefa N et al./2012 [48]	Oromia	Cohort	956	28.3	Low risk
Nekatibeb G 2007 [49]	Oromia	Cross-sectional	1832	8.6	Low risk
Kebede B et al./2013 [89]	Amhara	Retrospective cohort	416	21.4	Low risk
Eshete A et al./2013 [41]	Amhara	Cross-sectional	266	12.8	Low risk
Gebremedhin S et al./2012 [68]	SNNPR	Prospective cohort	575	16.5	Low risk
Mulatu H /2015 [63]	Addis Ababa	Cross-sectional	457	8.8	Low risk
Teshome D et al. /2006 [42]	Amhara	Cross-sectional	810	15.4	Low risk
Asefa M /2004 [50]	Oromia	Prospective Cohort	7586	10.0	Low risk
Mekbib TA/1995 [64]	Addis Ababa	Cross-sectional	7170	28.0	Low risk
Edris M /1996 [43]	Amhara	Cross-sectional	393	17.8	Low risk
Mengesha GH et al./2017 [57]	Tigray	Cross-sectional	1152	10.5	Low risk
Gebremedhin M et al./2015 [58]	Tigray	Cross-sectional	308	14.6	Low risk
Teklehaimanot N et al./2014 [59]	Tigray	Cross-sectional	520	8.1	Low risk
Sheferaw T/1990 [51]	Oromia	Cross-sectional	1707	12.3	Low risk
Gebregzabiherher Y et al./2017 [60]	Tigray	Cross-sectional	424	10.0	Low risk
Zeleke BM et al./2012 [44]	Amhara	Cross-sectional	305	17.1	Low risk
Gessessew A/2007 [61]	Tigray	Cross-sectional	7249	11.2	Low risk
Zerfu TA et al./2016 [52]	Oromia	Prospective cohort	374	9.1	Low risk
Tema T/2006 [53]	Oromia	Cross-sectional	645	22.5	Low risk
Korsak VS/1989 [30]	Addis Ababa	Cross-sectional	1106	55.9	Low risk
Wado YD et al./2014 [54]	Oromia	Prospective cohort	537	17.9	Low risk
Mekonen HK et al./2015 [62]	Tigray	Prospective cohort	1516	54.0	Low risk
Feleke Y/1999 [65]	Addis Ababa	Cross-sectional	4206	9.1	Low risk
Tilahun T et al./2015 [55]	Oromia	Prospective cohort	576	34.2	Low risk
Enquoselassie F /2000 [66]	Addis Ababa	Cross-sectional	9950	8.4	Low risk
Madebo T/1994 [29]	SNNPR	Cross-sectional	1260	8.0	Low risk
Demelash H et al. /2015 [56]	Oromia	Case-control	387	__	Low risk
Yesuf A et al./2016 [46]	Amhara	Case-control	453	__	Low risk
Desalegn L/2015 [45]	Amhara	Case-control	441	__	Low risk

### Quality of studies

The JBI quality appraisal criteria established for cross-sectional, case-control, and cohort studies were used. The studies included in this systematic review and meta-analysis had no considerable risk. Therefore, all the studies were considered [29, 38–72] (Table 1).

### Meta-analysis

#### Publication bias

**Fig. 2:**
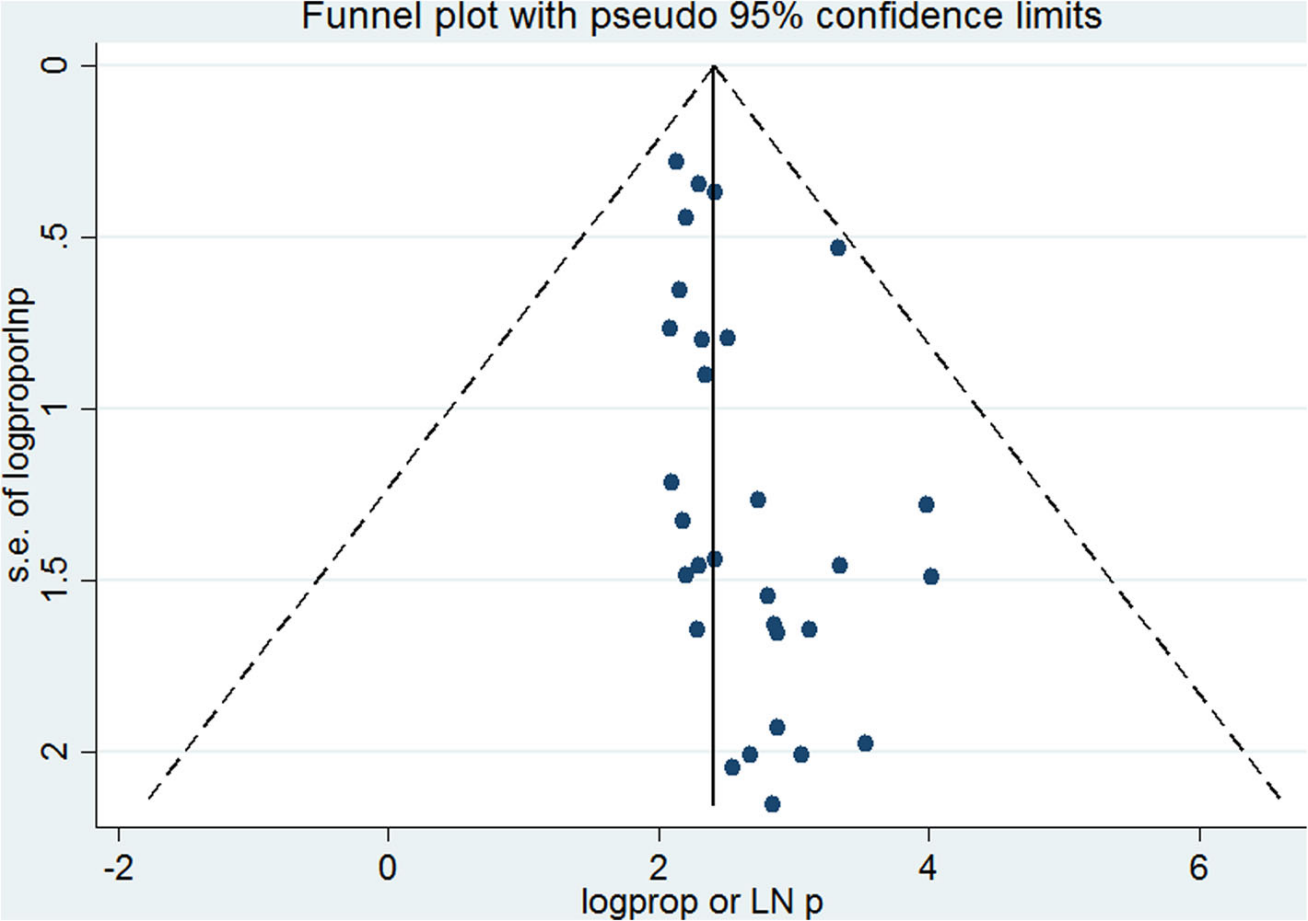
Funnel plot for publication bias, Logprop or LNP (log of proportion) represented in the x-axis and standard error of log proportion in the y-axis

A funnel plot showed a symmetrical distribution (Fig. 2). Egger’s regression test *p*-value was 0.063, which indicated the absence of publication bias.

#### Prevalence of low birth weight

**Fig. 3:**
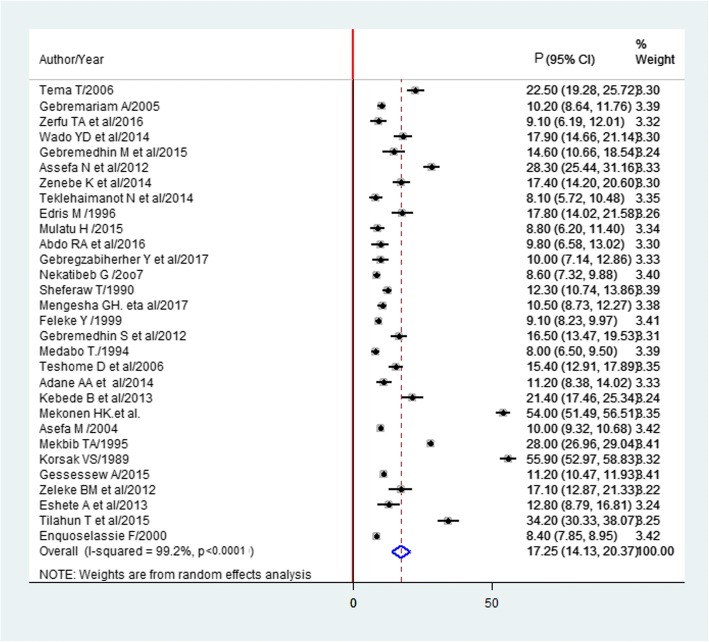
Forest plot of the prevalence of LBW with corresponding 95% CIs

The estimated overall prevalence of LBW is presented in a forest plot (Fig. 3). The overall prevalence of LBW was 17.3% (95% CI; 14.1–20.4; I^2^ = 99.2%).

#### Subgroup analysis

The subgroup analysis based on the region, study design, and year was done. Based on this, the prevalence of LBW found to be 22% in Addis Ababa, 23% in the cohort studies, and 17.7% between the year 2010 and 2017 (Table 2).

**Table 2:** The pooled prevalence of LBW, 95% CI and heterogeneity estimate with a *p*-value for the subgroup analysis

Variables	Characterstics	Pooled prevalence (95%CI)	I^2^ (*P*-value)
By region	Addis Ababa	22% (11.31–32.63)	99.8% (< 0.001)
	Tigray	18.1% (6.6–29.6)	99.5% (< 0.001)
	Oromia	16.8% (12.9–20.7)	97.8% (< 0.001)
	Amhara	16.0% (13.5–18.5)	73.4% (0.001)
	SNNPR	1.3% (6.2–16.4)	91.8% (< 0.001)
By study design	Cross-sectional	14.9%(11.6–18.1)	99.0% (< 0.001)
	Cohort	23.9 (12.3–35.5)	99.5%(< 0.001)
By year of publication	1989–2000	21.8% (11.2–32.4)	99.7% (< 0.001)
	2000–2010	11.7% (10.0–13.4)	95.1% (< 0.001)
	2010–2017	17.7% (11.5–23.9)	98.7%(< 0.001)

#### Sensitivity analysis

**Fig. 4:**
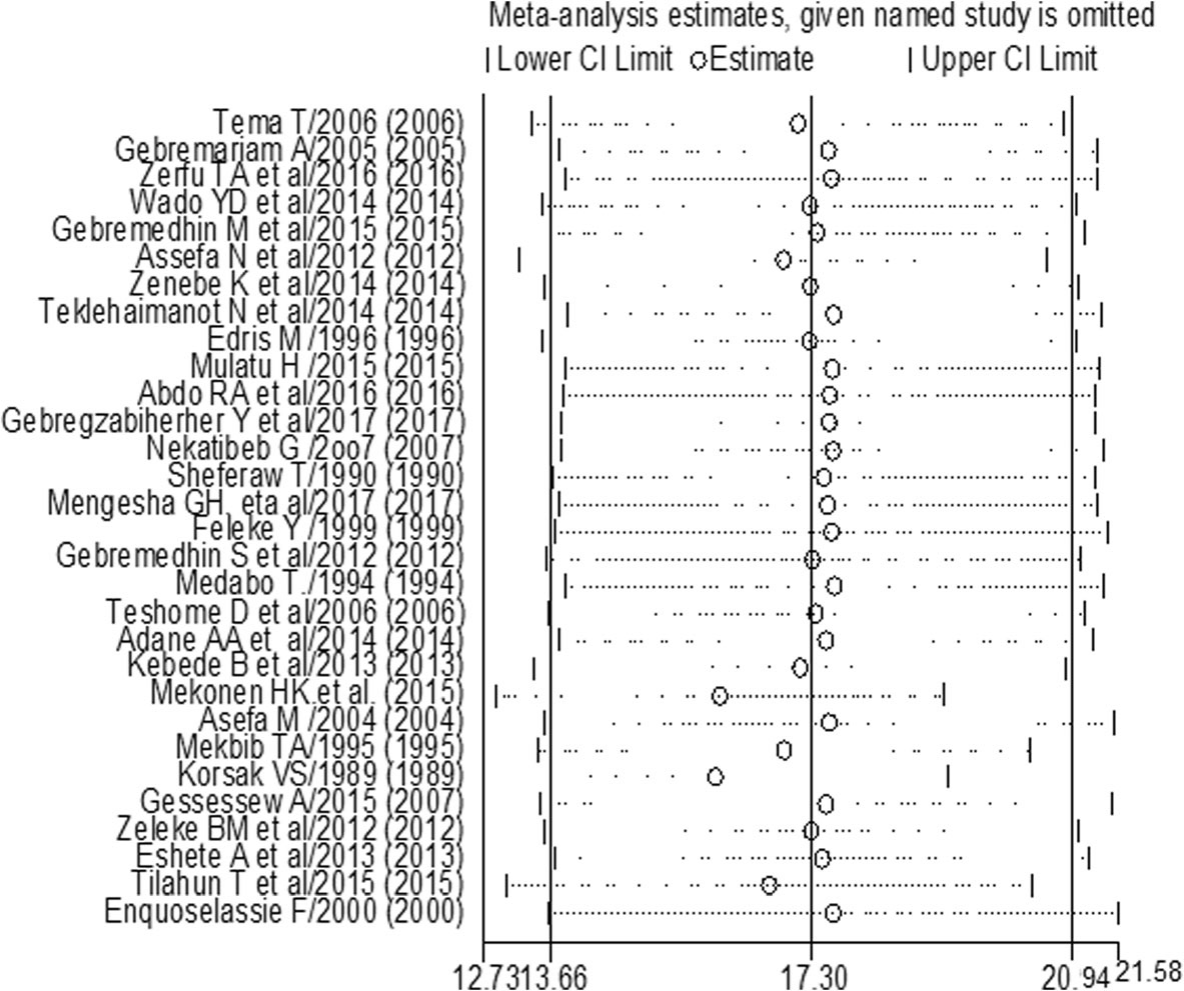
The sensitivity analysis showed the pooled prevalence when the studies omitted step by step

Mekonen HK et al. [62] and Korsak Vs [30] had shown an impact on the overall estimation (Fig. 4).

#### Time-trend analysis

**Fig. 5:**
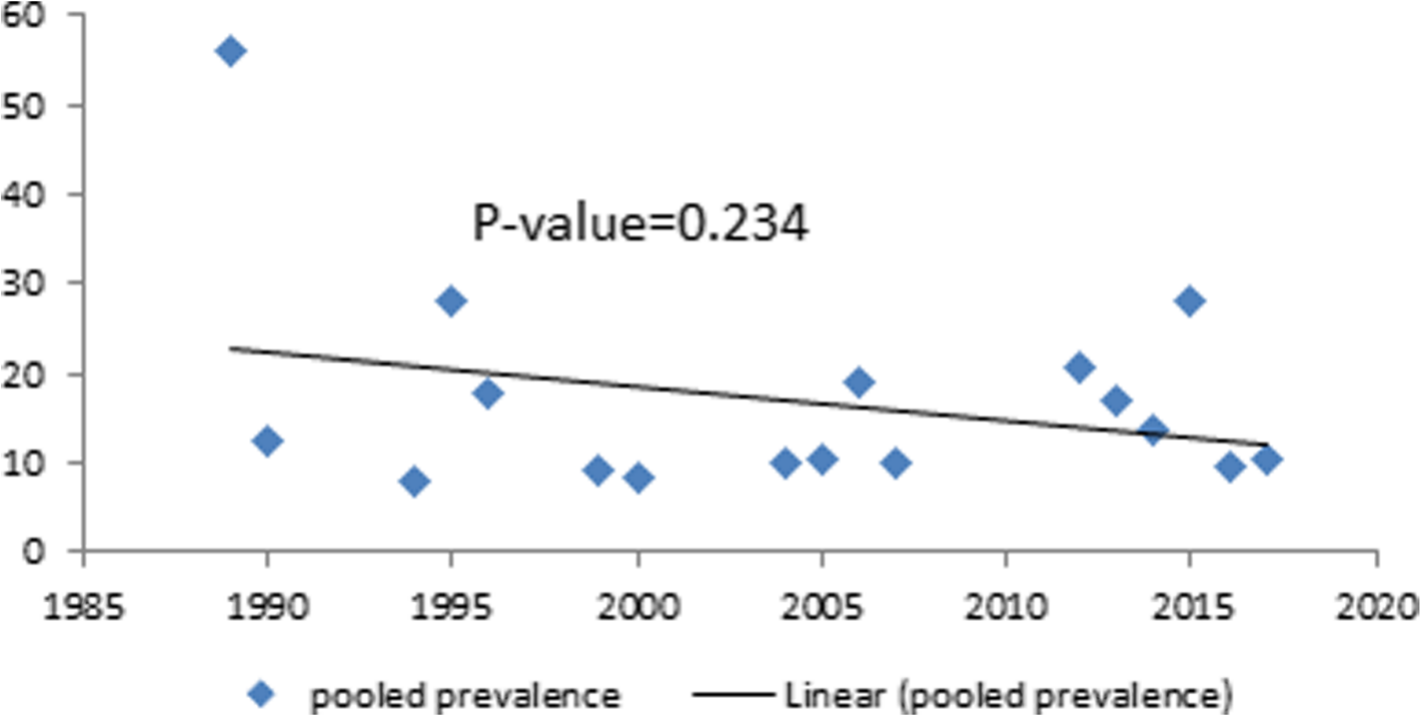
Time-trend analysis of the pooled prevalence of low birth weight in Ethiopia from 1989 to 2017

The time-trend analysis showed that the prevalence of LBW is decreased from 55.9% in 1989 to 10.4% in 2017. However, the pooled prevalence from year to year is not decreasing significantly (*p*-value = 0.234) (Fig. 5).

#### Associated factors

Based on this review, LBW in Ethiopian context is associated with socio-economic, obstetric and maternal behavior, infant, and environmental-related factors.

#### Socio-economic-related factors

Infants born from unmarried women (AOR = 10.5; 95% CI: 1.5–75.3) compared to married women [41], daily laborer women (AOR = 5.4;955 CI: 1.1–25.6) [46], and merchant (AOR = 0.1; 95 %CI = 0.02–0.52) [56] women as compared to house wife women were more likely to be LBW. Infants born from women who were on moderately or severely food insecure (AOR = 3.3; 95%CI: 1.0–10.3) [46], monthly family income ≤300 Ethiopian birr (AOR = 19.6; 95% CI:1.6–243.0) [41], and < 26 United States Dollarr (AOR = 3.8; 95% CI:1.5–9.4) [56] were associated with LBW. Besides, infant born from mothers with lack of formal education (AOR = 6; 95% CI: 1.3–26.9) [56] were higher risk to LBW.

**Fig. 6:**
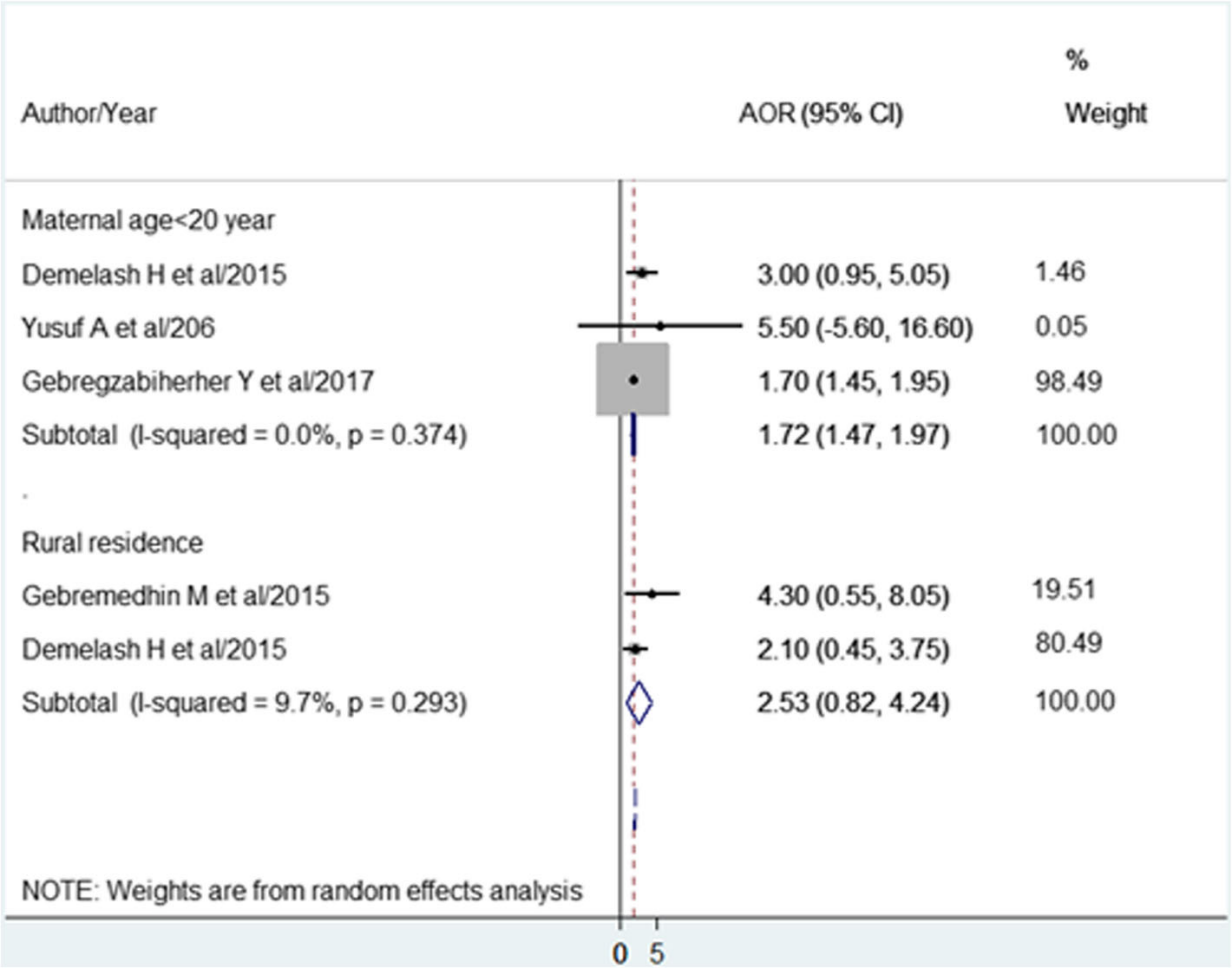
The pooled effects of maternal age < 20 years and rural residence on LBW of infants

The pooled effects of three studies [46, 56, 60] showed maternal age < 20 years was a significant associated factors, wheras the pooled effects of the other two studies [56, 58] found residence of mother had no significant association with LBW (Fig. 6).

#### Obstetiric and maternal behavior-related factors

Malaria attack during pregnancy (AOR = 4.9; 95%CI: 1.95–12.3) [38], history of delivering preterm or small baby (AOR = 8.4; 95% CI 2.4–29.4) [39], mothers who had no history of UTI and/or any documentation for bacteriuria during the current pregnancy (AOR = 0.1; 95% CI: 0.02–0.36) [41], women who had pregnancy complication (AOR = 4.39; 95%CI: 2.56–7.52) [46], presence of any chronic medical illness (AOR = 5.3, 95% CI:1.12–25.45), and mothers with a history of abortion 2.423 (1.744–15.317) [60] were associated with LBW.

Infants born from mothers with normal hemoglobin (AOR = 0.017; 95% CI: 0.002–0.176) [60] were less likely to be LBW, whereas infants born from maternal anemia (AOR = 5.52; 95%CI: 1.62–15.85) [40] were more likely to be LBW.

Maternal weight < 50 kg (AOR = 2.26; 95% CI = 1.06–4.80) [58] and maternal height < 1.5 m (AOR = 3.7; 95% CI = 1.22–11.28) [63] were also associated factors of LBW.

Primipara (AOR = 5.68; 95% CI: 2.20,14.66) [44] and unwanted pregnancies (AOR = 4.04, 95% CI:1.17–13.90) [59] were positively associated with LBW, where as mothers whose pregnancy was desired (AOR = 0.027; 95%CI: 0.004–0.207) [60] were less likely to deliver LBW infants.

Those mothers taking extra meal during pregnancy (AOR = 0.249; 95%CI = 0.064–0.960) [63] were less likely to deliver LBW infants.

Baseline maternal CD4 counts below 200 cells/mm3 (AOR = 4.24; 95% CI: 1.85–9.69), and 201–350 CD4 (AOR = 1.13; 95% CI: 0.53–2.37) as compared to> 350, and maternal exposure to Highly Active Antiret-roviral Treatment (HAART) (AOR = 8.26; 95% CI: 2.53–14.34) [40] were associated factors of LBW.

One research showed [56] that those infants born from mohers who had history of khat chewing were higher risk (AOR = 6.4; 95% CI = 2.42–17.10) to be LBW infants.

**Fig. 7:**
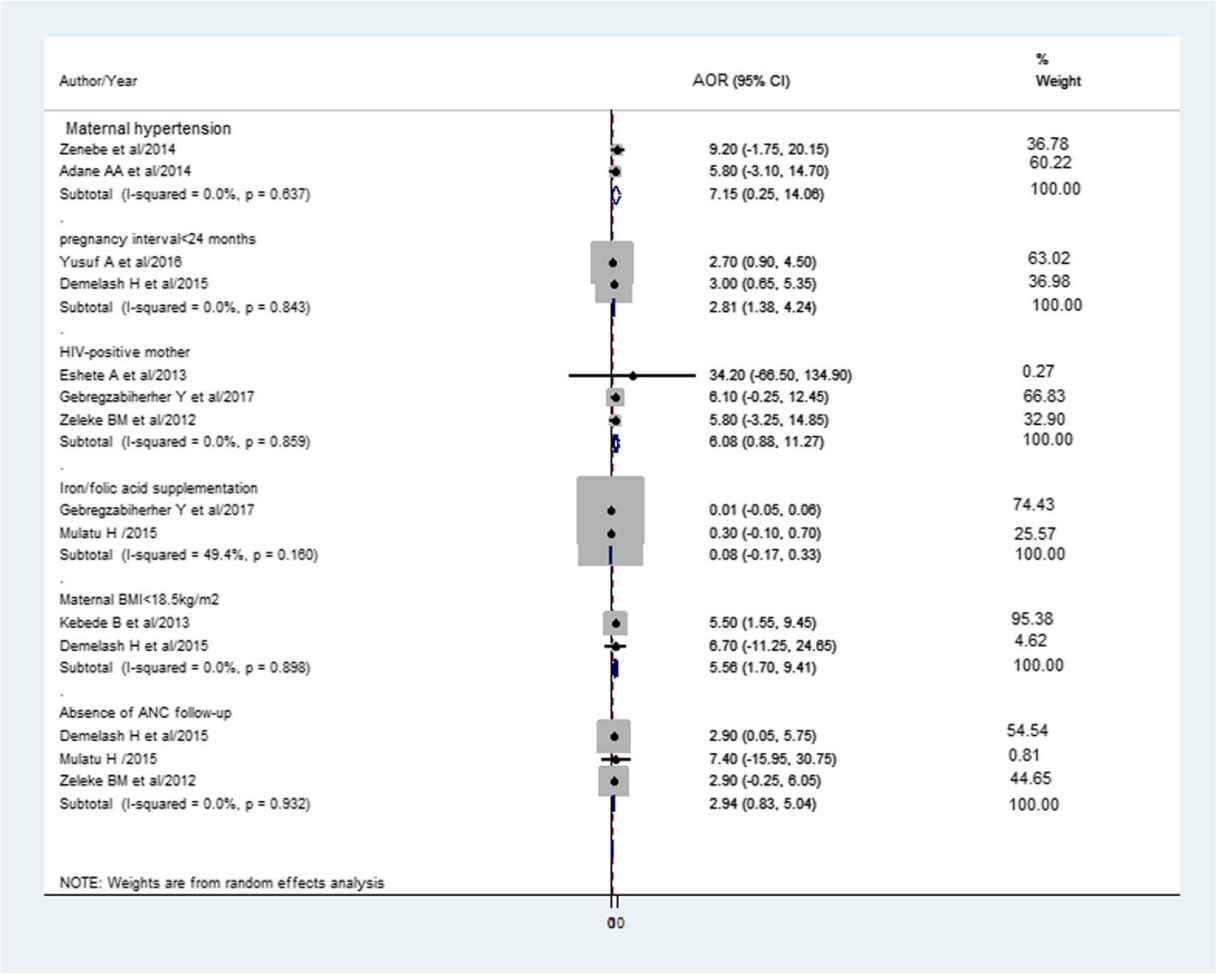
The pooled effects of maternal hypertension, pregnancy interval < 24 months, HIV positive mothers, iron/folic acid supplementation, maternal BMI < 18.5 kg/m^2^, and absence of ANC follow-up on LBW

The pooled effect of maternal hypertension [38, 39], being HIV positive mother [41, 44, 60], iron and/or folic acid supplementation [60, 63], and absence of antenatal care [44, 56, 63] were not became significantly associated factors. Inter-pregnancy interval less than 24 months [46, 56] and maternal body mass index < 18.5 kg/m2 [40, 56] were associated with LBW (Fig. 7).

#### Infant-related factors

Based on the pooled effects of two studies [38, 59], being female baby was not associated with LBW.

**Fig. 8:**
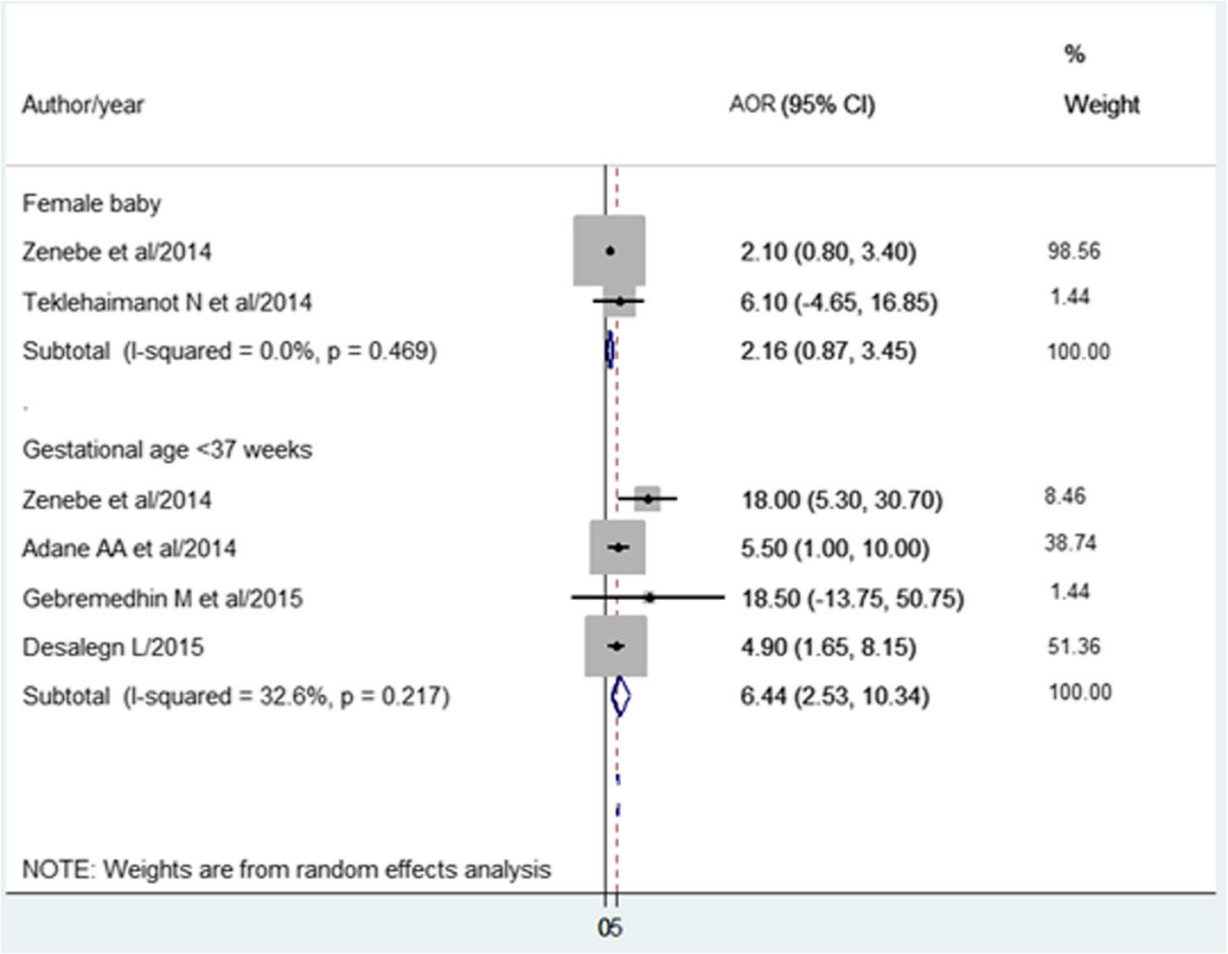
The pooled effects of sex of the baby and gestational age at birth on LBW

The pooled effects of four studies [38, 39, 45, 47] showed that gestational age less than 37 weeks was positively associated with LBW (Fig. 8). Another one study [44] showed infants’ gestational age 37–42 (AOR = 0.14; 95% CI: 0.04–0.47) and > 42 weeks (AOR = 0.13; 95% CI: 0.02,.68) were less likely to be born with LBW than < 37 weeks.

#### Environmental-related factors

Only one study [56] found, using firewood (AOR = 2.7; 95% CI: 1.01–7.17) and kerosene (AOR = 8.9; 95% CI: 2.54–31.11) for cooking, wash hands with water only (AOR = 2.2; 95% CI: 1.30–3.90), and not having separate kitchen room (AOR = 2.6; 95% CI: 1.36–4.85) were associated factors of LBW.

### Conceptual frame work of associated factors

**Fig. 9:**
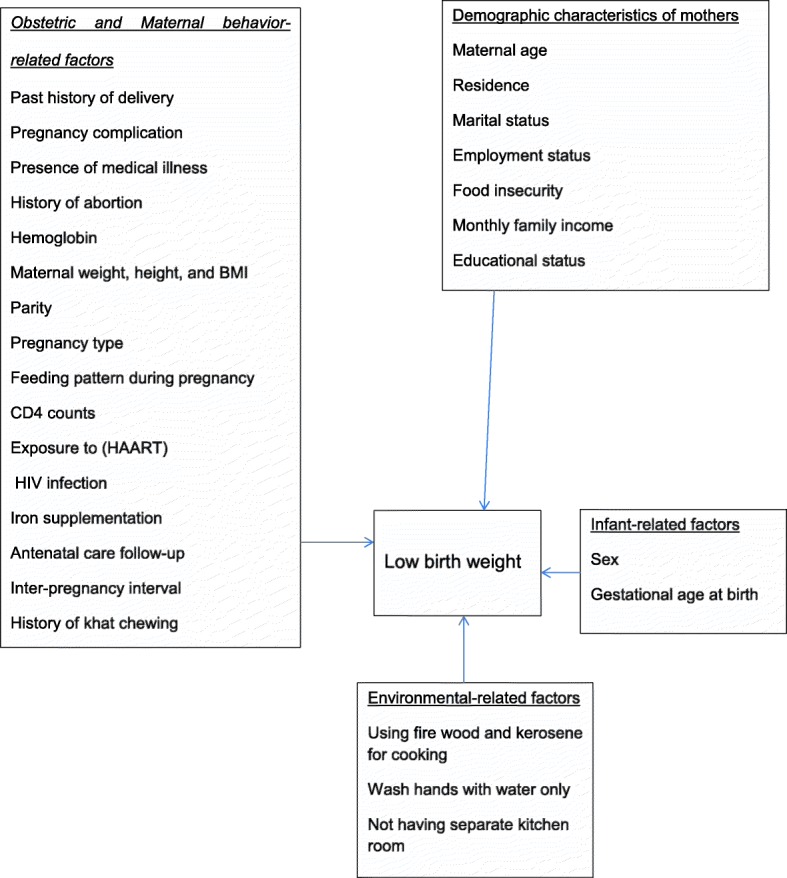
The summary presentation of associated factors of LBW in Ethiopia

Figure 9 shows the summary of associated factors of LBW.

## Discussion

The Millennium Development Goal-4 was aimed to reduce mortality of under-5 children by two-thirds [73]. It is continued to be one of the targets of Sustainable Development Goals; By 2030, all countries need to reduce neonatal mortality to below 12 per 1000 live births. For sub-Saharan Africa, the United Nation estimated 1 child among 12 dies before his or her fifth birthday that showed a great difference from developed countries, in which 1 dies among 147 children [74]. LBW is one of the leading causes of neonatal morbidity and mortality.

This meta-analysis was estimated the national prevalence of LBW in Ethiopia. Accordingly, the national pooled prevalence of LBW was 17.7% (14.1–20.4%). This result was higher than studies conducted in Indonesia (12.9%), Armenia (9.0%), and Tanzania (13.9%) [8]. This might be due to high prevalence of home delivery, in which many of the newborn weight might not be recorded, high prevalence of teenage delivery with a lower physical and psychological preparedness for their pregnancy, and lack of full ANC coverage, in which services provided, like health education about nutrition, getting iron supplementation, immunization, reassurance to get mental comfort influence the weight of newborns in Ehiopia. Besides, the high prevalence of preterm delivery contributes to the high prevalence of LBW in Ethiopia.

Furthermore, the time-trend analysis showed that the burden of LBW in Ethiopia was not decreased significantly. This might be due to a slow progress in the quality of healthcare services, like institutional delivery, postnatal services, ANC coverages, and immunization coverage still need further improvement. Moreover, many of the people are in low economic status that has impacts on maternal nutritional status before and during pregnancy and birth complication would be apparent as a result.

Based on the subgroup analysis, the prevalence of LBW was high in Addis Ababa as compared to other regions. Those studies included in this meta-analysis were conducted in governmental hospitals and data from private hospitals didn’t found. In Addis Ababa, there many private clinics and hospitals are avail. The probability of those mothers who had middle and high economic income delivered at private clinics would be high. As a result, the prevalence of LBW might vary as well.

Based on the pooled analysis of two or more AOR of studies, maternal age < 20 years, pregnancy interval < 24 months, maternal BMI < 18.5 kg/m^2^, and gestational age of the baby at birth < 37 weeks (preterm delivery) were associated with LBW.

The odds of infants born from mothers whose age < 20 years were nearly two times more risk to have LBW. This finding supported by other studies conducted in Nigeria [75] and Brazil [76]. This age group might be teenage pregnancy that mostly gives LBW infants usually due to unplanned and/or unwanted pregnancy, which leads to less attention to nutritional value and health care services utilization [77], lack of fair jobs, and a low level of knowledge of pregnancy’s danger sign and major risk factors [78] might be attributed to LBW in teenage pregnancy. Besides, pregnancy at this age might leads to stigma, social, and cultural malpractices during pregnancy and/or delivery time [79].

The odds of infants born within < 24 months birth interval were nearly three times to have LBW. This finding was in lined with a study in Sudan [11], Qatar [80], Iran [81], China [82], and United States [83]. The possible explanation might be due to short birth interval could result inadequate replenishment of maternal nutrients due to the physiologic depletion of folate that occurs in pregnancy and lactation, which result a negative impact on the growth of the fetus.

Those mothers who had BMI < 18.5 kg/m^2^ were more than five times risk to give LBW infants. Studies from Japan [84], China [85], and Thailand [86] are inagreement with the current finding. Mothers’ BMI is one of the best parameters to assess nutritional status. Those mother who had BMI < 18.5 kg/m^2^ indicates the presence of chronic malnutrition, in which fetal growth might be impaired. Therefore, LBW could be easily happen. Poor maternal nutritional status during pregnancy has also been associated with reduced placental weight and surface area, which could limit nutrient transfer from the placental circulation to the fetus. Moreover, undernutrition of mothers might leads to a reduction of serum concentrations of hormones, like leptin and estrogen, which results fetal growth impairment.

The odds of being premature birth was six times higher risk to be below normal birth weight. The finding from another study in India [87], Iran [12], and Brazil [76] also showed the positive association between premature birth and LBW. The several organ systems of human fetus usually become mature by the end of 37 weeks of gestation [88]. Therefore, those infants born before 37 weeks would have LBW more than that of babies born after 37 weeks of gestation.

This study identified important risk factors with independent effects on the burden of LBW in Ethiopia. National and regional policy and decision makers have work to improve these specific risk factors. Different strategies with appropriate community-based interventions on these factors need to be considered for improving overall child health in Ethiopia.

### Strength and limitation

This systematic review and meta-analysis is the national estimation conducted in Ethiopia. It may be lacked national representativeness because no data were found from Benishangul Gumuz, Afar, Gambella, Somalia, Dire Dawa, and Harare regions. Time-trend analysis might not reflect the exact trend because all the years didn’t have reported data.

## Conclusions

The prevalence of low birth weight in Ethiopia remains high. This review may help policy-makers and program officers to design interventions on preventing low birth weight.

## Abbreviations

ANC: Antenatal care
ART: Antiretroviral Therapy
LBW: Low Birth Weight
OR: Odds Ratio
SNNPR: Southern Nation Nationality and Peoples Region

## References

[1] Rezende Chrisman J, Mattos IE, Koifman RJ, Koifman S, Moraes Mello Boccolini P, Meyer A (2016). Prevalence of very low birthweight, malformation, and low Apgar score among newborns in Brazil according to maternal urban or rural residence at birth. J Obstet Gynaecol Res.

[2] Azoumah K, Djadou K, Aboubakari A, Bothon A, Agbodjan-Djossou O, Agbèrè A.General medicine: open evaluation of the glycemia of low-weight newborns within the 24th hour of life at Lome University hospital (Togo). Archives de pediatrie: organe officiel de la Societe francaise de pediatrie. 2011;18(10):1037–1043. 10.1016/j.arcped.2011.07.012 21868207

[3] Seyum T, Ebrahim E. Proportion of neonatal hypothermia and associated factors among new-borns at Gondar University teaching and Refferal hospital, Northwest Ethiopia: a hospital based cross sectional study. General Medicine: Open Access. 2015;2015.

[4] Hack Maureen, Klein Nancy K., Taylor H. Gerry (1995). Long-Term Developmental Outcomes of Low Birth Weight Infants. The Future of Children.

[5] Gutbrod T, Wolke D, Soehne B, Ohrt B, Riegel K (2000). Effects of gestation and birth weight on the growth and development of very low birthweight small for gestational age infants: a matched group comparison. Arch Dis Child Fetal Neonatal Ed.

[6] WHO (2014). World health ranking: Ethiopia low birth weight.

[7] WHO. WHA Global Nutrition Targets 2025: Low Birth Weight Policy Brief. 2014.

[8] Mahumud RA, Sultana M, Sarker AR (2017). Distribution and determinants of low birth weight in developing countries. J Prev Med Public Health.

[9] Milabyo KP (2006). Low birth weight in Maniema (Democratic Republic of Congo). Sante (Montrouge, France).

[10] Ugboma H, Onyearugha C (2013). Low birthweight delivery: prevalence and associated factors as seen at a tertiary health facility. Niger J Clin Pract.

[11] Adam I, Ismail MH, Nasr AM, Prins MH, Smits LJ (2009). Low birth weight, preterm birth and short interpregnancy interval in Sudan. J Matern Fetal Neonatal Med.

[12] Bazyar J, Daliri S, Sayehmiri K, Karimi A, Delpisheh A (2015). Assessing the relationship between maternal and neonatal factors and low birth weight in Iran; a systematic review and meta-analysis. J. Med. Life.

[13] Ndiaye O, Diallo D, Bâ MG, Diagne I, Moreau J-C, Diadhiou F (2002). Maternal risk factors and low birth weight in Senegalese teenagers: the example of a hospital Centre in Dakar. Cahiers d'études et de recherches francophones. Santé.

[14] Owusu JT, Anderson FJ, Coleman J, Oppong S, Seffah JD, Aikins A (2013). Association of maternal sleep practices with pre-eclampsia, low birth weight, and stillbirth among Ghanaian women. Int J Gynecol Obstet.

[15] Valea I, Tinto H, Drabo MK, Huybregts L, Sorgho H, Ouedraogo J-B (2012). An analysis of timing and frequency of malaria infection during pregnancy in relation to the risk of low birth weight, anaemia and perinatal mortality in Burkina Faso. Malar J.

[16] Oraneli BU, Okeke OC, Ubachukwu PO (2013). Effect of placental malaria on birth weight of babies in Nnewi, Anambra state Nigeria. J. Vector Borne Dis.

[17] Njim T, Atashili J, Mbu R, Choukem S-P (2015). Low birth weight in a sub-urban area of Cameroon: an analysis of the clinical cut-off, incidence, predictors and complications. BMC pregnancy and childbirth.

[18] Oladeinde HB, Oladeinde OB, Omoregie R, Onifade AA (2015). Prevalence and determinants of low birth weight: the situation in a traditional birth home in Benin City Nigeria. Afr Health Sci.

[19] Feresu SA, Harlow SD, Woelk GB (2015). Risk factors for low birthweight in Zimbabwean women: a secondary data analysis. PLoS One.

[20] Mabiala-Babela J, Matingou V, Senga P (2007). Risk factors for low birth weight in Brazzaville, Congo. J. Gynecol. Obstet. Biol. Reprod.

[21] Kaye DK, Mirembe FM, Bantebya G, Johansson A, Ekstrom AM (2006). Domestic violence during pregnancy and risk of low birthweight and maternal complications: a prospective cohort study at Mulago hospital, Uganda. Tropical Med Int Health.

[22] Awoleke J (2012). Maternal risk factors for low birth weight babies in Lagos, Nigeria. Arch Gynecol Obstet.

[23] Haggaz AD, Radi EA, Adam I (2010). Anaemia and low birthweight in western Sudan. Trans R Soc Trop Med Hyg.

[24] Rwebembera AA-B, Munubhi E, Manji K, Mpembeni R, Philip J (2005). Relationship between infant birth weight≤ 2000 g and maternal zinc levels at Muhimbili National Hospital, Dar Es Salaam, Tanzania. J Trop Pediatr.

[25] Muchemi OM, Echoka E, Makokha A. Factors associated with low birth weight among neonates born at Olkalou District hospital, central region, Kenya. Pan Afr. Med. J. 2015;20(1). 10.11604/pamj.2015.20.108.4831 PMC4458305 26090056

[26] Dreyfuss ML, Msamanga GI, Spiegelman D, Hunter DJ, Urassa EJ, Hertzmark E (2001). Determinants of low birth weight among HIV-infected pregnant women in Tanzania. Am J Clin Nutr.

[27] Sadissou I, d’Almeida T, Cottrell G, Luty A, Krawice-Radanne I, Massougbodji A (2014). High plasma levels of HLA-G are associated with low birth weight and with an increased risk of malaria in infancy. Malar J.

[28] Mombo-Ngoma G, Honkpehedji J, Basra A, Mackanga JR, Zoleko RM, Zinsou J (2017). Urogenital schistosomiasis during pregnancy is associated with low birth weight delivery: analysis of a prospective cohort of pregnant women and their offspring in Gabon. Int J Parasitol.

[29] Madebo T (1994). A two year retrospective study of birth weight in Sidamo regional hospital. Ethiop Med J.

[30] Korsak VS (1989). Incidence and some perinatal problems of multiple pregnancies in a central referral hospital Addis Ababa. Ethiop Med J.

[31] Moher D, Liberati A, Tetzlaff J, Altman DG, Group P (2009). Preferred reporting items for systematic reviews and meta-analyses: the PRISMA statement. PLoS Med.

[32] Peters M, Godfrey C, McInerney P, Soares C, Hanan K, Parker D (2015). The Joanna Briggs institute Reviewers' manual 2015: methodology for JBI scoping reviews.

[33] World Health Organization. Global Nutrition Targets 2025: Low birth weight policy brief. Geneva: 2014. Available at https://www.who.int/nutrition/publications/globaltargets2025_policybrief_lbw/en/.

[34] Egger M, Smith GD, Schneider M, Minder C (1997). Bias in meta-analysis detected by a simple, graphical test. BMJ.

[35] Ioannidis J (2008). Interpretation of tests of heterogeneity and bias in meta-analysis. J Eval Clin Pract.

[36] Higgins J, Thompson SG (2002). Quantifying heterogeneity in a meta-analysis. Stat Med.

[37] Borenstein M, Hedges LV, Higgins J, Rothstein HR (2010). A basic introduction to fixed-effect and random-effects models for meta-analysis. Res Synth Methods.

[38] Zenebe K, Awoke T, Birhan N (2014). Low birth weight & associated factors among newborns in Gondar town, north West Ethiopia: institutional based cross-sectional study. Indo Global J. Pharm.

[39] Adane AA, Ayele TA, Ararsa LG, Bitew BD, Zeleke BM (2014). Adverse birth outcomes among deliveries at Gondar University hospital, Northwest Ethiopia. BMC pregnancy and childbirth.

[40] Kebede B, Andargie G, Gebeyehu A (2013). Birth outcome and correlates of low birth weight and preterm delivery among infants born to HIV-infected women in public hospitals of Northwest Ethiopia. Health.

[41] Eshete A, Birhanu D, Wassie B (2013). Birth outcomes among laboring mothers in selected health facilities of north Wollo zone, Northeast Ethiopia: a facility based cross-sectional study. Health.

[42] Teshome D, Telahun T, Solomon D (2006). Abdulhamid I. a study on birth weight in a teaching-referral hospital, Gondar, Ethiopia. Cent. Afr. J. Med.

[43] Edris M, Erakli G (2017). The prevalence of low birth weight and factors associated with low birth weight delivery in Gondar region, north West Ethiopia. Ethiop. J. Heal. Dev.

[44] Zeleke BM, Zelalem M, Mohammed N. Incidence and correlates of low birth weight at a referral hospital in Northwest Ethiopia. Pan Afr. Med. J. 2012;12(1). PMC3396870 22826729

[45] Desalegn L (2015). Determinants of low birth weight in Debre Berhan referral hospital, north Shoa zone, Amhara regional state, Ethiopia; a case-control study.

[46] Aman Yesuf AY, Fikre Enquoselassie FE, Seifu Hagos SH, MA MA. Effect of Interpregnancy interval on low birth weight in Gondar and Bahir Dar referral hospital: a case control study from north West Ethiopia. Journal of Health, Medicine and Nursing. 2016:31.

[47] Gebremariam A (2005). Factors predisposing to low birth weight in Jimma hospital south western Ethiopia. East Afr Med J.

[48] Assefa N, Berhane Y, Worku A (2012). Wealth status, mid upper arm circumference (MUAC) and antenatal care (ANC) are determinants for low birth weight in Kersa, Ethiopia. PLoS One.

[49] Nekatibeb G (2007). Analysis of birth weight in Metu Karl hospital: south West Ethiopia. Ethiop Med J.

[50] Asefa M, Tessema F. Patterns of birth weight at a community level in Southwest Ethiopia. Ethiopian Journal of Health Sciences. 2004;14(1).

[51] Sheferaw T (1990). Some factors associated with birth weight in Jima southwestern Ethiopia. Ethiopian medical journal.

[52] Zerfu TA, Umeta M, Baye K (2016). Dietary diversity during pregnancy is associated with reduced risk of maternal anemia, preterm delivery, and low birth weight in a prospective cohort study in rural Ethiopia. Am J Clin Nutr.

[53] Tema T (2006). Prevalence and determinants of low birth weight in Jimma zone, Southwest Ethiopia. East Afr Med J.

[54] Wado YD, Afework MF, Hindin MJ (2014). Effects of maternal pregnancy intention, depressive symptoms and social support on risk of low birth weight: a prospective study from southwestern Ethiopia. PLoS One.

[55] Tilahun T, Araya F, Tura G (2015). Perinatal complications of twin deliveries at Jimma University specialized hospital, Southwest Ethiopia: a facility-based cohort study. Science, Technology and Arts Research Journal.

[56] Demelash H, Motbainor A, Nigatu D, Gashaw K, Melese A (2015). Risk factors for low birth weight in bale zone hospitals, south-East Ethiopia: a case–control study. BMC pregnancy and childbirth..

[57] Mengesha HG, Wuneh AD, Weldearegawi B, Selvakumar DL (2017). Low birth weight and macrosomia in Tigray, northern Ethiopia: who are the mothers at risk?. BMC Pediatr.

[58] Gebremedhin M, Ambaw F, Admassu E, Berhane H (2015). Maternal associated factors of low birth weight: a hospital based cross-sectional mixed study in Tigray, northern Ethiopia. BMC pregnancy and childbirth.

[59] Teklehaimanot N, Hailu T, Assefa H (2014). Prevalence and factors associated with low birth weight in Axum and Laelay Maichew districts, North Ethiopia: a comparative cross sectional study. Int J Nutr Food Sci.

[60] Gebregzabiherher Y, Haftu A (2017). The prevalence and risk factors for low birth weight among term newborns in Adwa general hospital. Northern Ethiopia.

[61] Gessessew A (2007). Twin deliveries in a zonal hospital: ten years retrospective study. Ethiop Med J.

[62] Mekonen HK, Nigatu B, Lamers WH (2015). Birth weight by gestational age and congenital malformations in northern Ethiopia. BMC pregnancy and childbirth.

[63] Mulatu H, Betre M (2015). Assessment of the magnitude and factors associated with low birth weight among newborns in public hospitals of Addis Ababa.

[64] Mekbib T (1995). Breech delivery and foetal outcome: a review of 291 cases. Ethiop Med J.

[65] Feleke Y, Enquoselassie F (1999). Maternal age, parity and gestational age on the size of the newborn in Addis Ababa. East Afr Med J.

[66] Enquoselassie F, Minyilshewa A (2000). Changes in birth-weight of hospital-delivered neonates in Addis Ababa. Ethiop J Health Dev.

[67] Abdo RA, Endalemaw TB, T FY. Prevalence and associated factors of adverse birth outcomes among women attended maternity Ward at Negest Elene Mohammed memorial general Hospital in Hosanna Town, SNNPR, Ethiopia. Journal of Womens Health Care. 2016;5(4).

[68] Gebremedhin S, Enquselassie F, Umeta M (2012). Independent and joint effects of prenatal zinc and vitamin a deficiencies on birthweight in rural Sidama, southern Ethiopia: prospective cohort study. PLoS One.

[69] Alemu T, Umeta M (2016). Prevalence and predictors of" small size" babies in Ethiopia: in-depth analysis of the Ethiopian demographic and health survey, 2011. Ethiopian journal of health sciences.

[70] Central Statistical Agency [Ethiopia]. Ethiopia demographic and health survey 2016 Addis Ababa, Ethiopia, and Rockville, Maryland. USA, CSA and ICF; 2017.

[71] Central Statistical Agency [Ethiopia] and ORC Macro (2006). Ethiopia demographic and health survey 2005.

[72] Central Statistical Authority [Ethiopia] and ORC Macro. Ethiopia demographic and health survey 2000. Maryland, USA: Addis Ababa, Ethiopia and Calverton. p. 2001.

[73] Lawn Joy E., Lee Anne CC, Kinney Mary, Sibley Lynn, Carlo Wally A., Paul Vinod K., Pattinson Robert, Darmstadt Gary L. (2009). Two million intrapartum-related stillbirths and neonatal deaths: Where, why, and what can be done?. International Journal of Gynecology & Obstetrics.

[74] UNICEF (2015). Levels and trends in child mortality:estimates developed by the UN inter-agency Group for Child Mortality Estimation.

[75] Takai IU, Bukar M, Audu BM (2014). A prospective study of maternal risk factors for low birth weight babies in Maiduguri, north-eastern Nigeria. Nigerian Journal of Basic and Clinical Sciences.

[76] CQdS M, BCdA C, Mandetta MA, MMFG B (2015). Low birth weight in a municipality in the southeast region of Brazil. Revista brasileira de enfermagem.

[77] Acharya DR, Bhattarai R, Poobalan A, Teijlingen VE, Chapman G (2014). Factors associated with teenage pregnancy in South Asia.

[78] Chowdhury A, Halder K, Haque I, Muhammad F, Hasan M (2017). Status of knowledge on the risk factors of low birth weight among the women of reproductive age in rural Bangladesh. Epidemiology (Sunnyvale).

[79] Kaphagawani NC, Kalipeni E (2017). Sociocultural factors contributing to teenage pregnancy in Zomba district, Malawi. Global public health.

[80] Bener A, Saleh NM, Salameh KMK, Basha B, Joseph S, Samson N (2012). The impact of the interpregnancy interval on birth weight and other pregnancy outcomes. Revista Brasileira de Saúde Materno Infantil.

[81] Mirahmadizadeh A, Soleimani A, Moradi F, Hesami E, Kasraeian M, Delam H (2017). Prevalence and risk factors of low birth weight in Fars province, south of Iran, 2014. Journal of health sciences and surveillance system.

[82] Zhang Q, Dang S, Bai R, Mi B, Wang L, Yan H (2018). Association between maternal interpregnancy interval after live birth or pregnancy termination and birth weight: a quantile regression analysis. Sci Rep.

[83] Gemmill A, Lindberg LD (2013). Short interpregnancy intervals in the United States. Obstet Gynecol.

[84] Murai U, Nomura K, Kido M, Takeuchi T, Sugimoto M, Rahman M. Pre-pregnancy body mass index as a predictor of low birth weight infants in Japan. Asia Pac J Clin Nutr. 2016. 10.6133/apjcn.032016.11 28429908

[85] Ronnenberg AG, Wang X, Xing H, Chen C, Chen D, Guang W (2003). Low preconception body mass index is associated with birth outcome in a prospective cohort of Chinese women. J Nutr.

[86] Sananpanichkul P, Rujirabanjerd S (2015). Association between maternal body mass index and weight gain with low birth weight in eastern Thailand. Southeast Asian J Trop Med Public Health.

[87] Ahankari A, Bapat S, Myles P, Fogarty A, Tata L. Factors associated with preterm delivery and low birth weight: a study from rural Maharashtra, India. F1000Research. 2017;6. 10.12688/f1000research.10659.1 PMC5428483 28529697

[88] Sands J, Dobbing J (1985). Continuing growth and development of the third-trimester human placenta. Placenta.

[89] Mengesha E, Airgecho T, Negera E, Kebede M (2015). Prevalence of triple viral infections of human immunodeficiency virus (HIV), hepatitis B and C among tuberculosis patients and associated risk factors: the case of west Arsi zone, Ethiopia. Afr J Microbiol Res.

